# A mixed methods study on the readiness of dental, medical, and nursing students for interprofessional learning

**DOI:** 10.1371/journal.pone.0255086

**Published:** 2021-07-22

**Authors:** Mitsuyuki Numasawa, Nobutoshi Nawa, Yu Funakoshi, Kanako Noritake, Jun Tsuruta, Chiharu Kawakami, Mina Nakagawa, Kumiko Yamaguchi, Keiichi Akita

**Affiliations:** 1 Institute of Education, Tokyo Medical and Dental University, Tokyo, Japan; 2 Department of Medical Education Research and Development, Tokyo Medical and Dental University, Tokyo, Japan; 3 Department of Tokyo Metropolitan Health Policy Advisement, Tokyo Medical and Dental University, Tokyo, Japan; 4 Oral Diagnosis and General Dentistry, Dental Hospital, Tokyo Medical and Dental University, Tokyo, Japan; 5 Gerontological Nursing, Graduate School of Nursing Science, St. Luke’s International University, Tokyo, Japan; 6 Department of Clinical Anatomy, Tokyo Medical and Dental University, Tokyo, Japan; International Medical University, MALAYSIA

## Abstract

**Background:**

Interprofessional education (IPE) is crucial in dentistry, medicine, and nursing. However, scant mixed methods studies have compared the IPE outcomes across these disciplines to develop evidence-based IPE. This study explored the differences in the readiness of dental, medical, and nursing students for interprofessional learning before and after IPE workshops and elucidated reasons for this disparity.

**Methods:**

Data were obtained from dental, medical, and nursing students who participated in IPE workshops conducted at Tokyo Medical and Dental University in Japan in 2019 and 2020. The participants filled the validated Japanese version of the Readiness for Interprofessional Learning Scale (RIPLS) before and after attending the workshops (n = 378). Paired t-tests were performed to assess differences between the pre- and post- workshop RIPLS scores. Welch’s t-tests were deployed to evaluate interdisciplinary differences in their scores. Qualitative analyses were conducted using an explanatory sequential design with focus group discussions (FGDs) held with 17 dental students to explain the quantitative results.

**Results:**

Total RIPLS scores increased significantly for every discipline after the workshops (*p* < 0.001). Dental students scored significantly lower pre- and post- workshop aggregates than medical and nursing students, respectively (*p* < 0.001). The FGDs yielded three principal themes in the explanations tendered by dental students on their lower scores: 1) dental students rarely felt the need for interprofessional collaborations, 2) dentists often worked without the need for interprofessional collaborations, and 3) dental students believed their contribution to the workshop was insufficient.

**Conclusions:**

The results revealed divergences in the readiness of dental, medical, and nursing students for interprofessional learning, and the study illuminated possible reasons for these disparities. These outcomes will help develop evidence-based IPE by indicating approaches to place a higher value on interprofessional collaborations in educational environments, ameliorate the awareness of educators, and enhance the workshop facilitation style.

## Introduction

Interprofessional education (IPE) is essential for healthcare students as it prepares them to collaborate effectively in multidisciplinary teams [1, 2]. The World Health Organization (WHO) constantly emphasizes the importance of IPE [1, 3], and the evidence accumulated by empirical studies suggests that IPE exerts a beneficial impact on students [4–7]. The disciplines of medicine and nursing are most frequently involved in IPE [8]. Conversely, students of dentistry were rarely engaged in IPE until the 2000s [9, 10]. However, the need to incorporate IPE into dental education is increasingly highlighted because systemic and oral health conditions interact, and dentistry discharges a critical role in the provision of primary care [10, 11]. Following this trend, IPE has made steady progress in dental education since the Interprofessional Education Collaborative (IPEC) published its core competencies in 2011 [12] and the Commission on Dental Accreditation (CODA) established two predoctoral accreditation standards relating to IPE [13–15]. It has become increasingly important in this historical context to develop and improve IPE programs and environments involving dental, medical, and nursing students.

The revised Biggs’ presage-process-product (3P) model for IPE is frequently employed to evaluate the effectiveness of IPE in both educational and research settings [8, 16, 17]. The “presage factors” denote the contexts in which teaching occurs and allude to the characteristics of teachers and learners [8, 17]. If this model is employed as a theoretical framework, these factors may vary across student disciplines. Therefore, the “product factors” referring to the collaborative outcomes of IPE [8, 17] may also vary across disciplines. Several studies have thus reported discipline-based differences in the impact of IPE on students [18–20]. Thus, it is necessary to compare the outcomes of IPE across disciplines to develop evidence-based IPE programs that enable students from multiple disciplines to achieve sufficient levels of interprofessional collaboration. Mixed methods studies are crucial to the achievement of this purpose because they allow the intensive examination of the factors and can illuminate the reasons for quantitative differences observed in the effects of IPE across disciplines by incorporating qualitative surveys [21]. However, mixed methods studies on IPE involving dental, medical, and nursing students are still lacking [22].

This study adopted the validated Japanese version of the Readiness for Interprofessional Learning Scale (RIPLS) to achieve its aim of exploring interdisciplinary differences in IPE outcomes observed in dental, medical, and nursing students [23, 24]. It also intended to elucidate the factors influencing the disparities and illuminate the reasons for the results obtained through quantitative analyses by conducting a mixed methods study using an explanatory sequential design [25]. The Tokyo Medical and Dental University (TMDU), located in Japan’s capital city, represents one of the few Japanese universities encompassing schools of medicine, dentistry, and nursing. This study was conducted at TMDU to take advantage of this rare feature.

## Materials and methods

### Sample

A two-day annual IPE workshop has been conducted at TMDU since 2012. The participants of the 2019 and 2020 editions of the workshop comprised dental, medical, nursing, medical technology, dental hygiene, and dental technician students from TMDU; pharmacy students from Hoshi University; and social work students from Waseda University and Sophia University in Japan. The workshops were compulsory for the students from TMDU; whereas, they were elective for the students from the other universities. All the participants were enrolled in the final year of their undergraduate program, except for some third-year social work students. A total of 680 students participated in the workshops in 2019 and 2020.

The present study’s research question was aimed at assessing the outcomes of IPE in dental, medical, and nursing students. Hence, it focused on the analyses of data obtained from dental, medical, and nursing students in 2019 and 2020. Dental hygiene and dental technician students were not included in this study. The validated Japanese version of RIPLS was filled in by 406 of 422 dental, medical, and nursing students [24] before and after attending the IPE workshops (pre- and post-test). Data pertaining to 28 students were excluded because of missing values in the pre- or post-tests. Ultimately, analyses were performed on data attained from 378 students.

### Ethical considerations

The study performed secondary analyses of data obtained for educational purposes. The participating students were informed of their right to refuse to allow researchers to use their data for secondary analyses. This study did not involve any minors or patients. This research project was approved by the Ethics Committee of the TMDU (M2000-1786), which waived the need for consent.

### Intervention

The principal segment of the IPE workshop entailed an interactive workshop using simulated clinical scenarios that were collaboratively prepared through interdisciplinary discussions representing faculty members across the educational specialties of all participants. Multiple and complex case-based problems were designed to adequately enable students from all the represented disciplines to fully utilize their expertise. The students were informed that the workshop proposed to increase their knowledge of divergent perspectives and roles of different disciplines so that they could collaborate effectively in multidisciplinary teams to provide the best possible healthcare to patients.

The participants were divided into multidisciplinary groups of seven to eight students. At the beginning of the workshop, all the students introduced themselves and explained their disciplines and professions to the other members of their groups. A total of around four hours were expended over the two workshop days on group discussions and group work pertaining to the scenarios. Students were assigned tasks of discussing how to solve multiple problems collaboratively. Every group delivered a team presentation on each day after the group discussions, followed by a question-and-answer session.

The simulated scenarios were presented through the learning management system WebClass (DATA PACIFIC (JAPAN) LTD, Kunitachi, Tokyo, Japan). Until 2019, the workshops were held in lecture rooms at TMDU; whereas, the 2020 workshop was held using the video teleconference software Zoom, version 5.1.0 (Zoom Video Communications, Inc., San Jose, CA, USA), during the coronavirus disease (COVID-19) pandemic. The Breakout Rooms of Zoom were utilized for group works. The online presentation software Google Slides (Google LLC, Mountain View, CA, USA) were also utilized for writing and sharing the opinions and ideas of the students. The goals and objectives of the workshop set for the students and shared with the faculty, as well as the tasks assigned to them were the same in 2019 and 2020.

### Quantitative analyses

Participants completed the validated Japanese version of RIPLS [24] just before and immediately after the IPE workshops (pre- and post-test). This instrument comprises 19 items measuring the readiness of respondents for interprofessional learning on a five-point Likert-like scale ranging from “strongly agree (five)” to “strongly disagree (one).” Some reverse-scored items are included in the scale. Higher scores indicate better readiness for interprofessional learning. The Cronbach’s alpha value computed for RIPLS in this study was 0.87, indicating sufficient internal consistency.

Two-tailed paired t-tests were employed to assess differences between the pre- and post- workshop RIPLS scores of each discipline represented by the students. Two-tailed Welch’s t-tests were performed to evaluate interdisciplinary differences observed in the pre-test scores, the post-test scores, and the changes in the scores after the workshops. All statistical analyses were conducted using RStudio, version 1.1453 (RStudio, Inc., Boston, MA, USA). The results were considered statistically significant at *p* < 0.05.

### Qualitative analyses

Qualitative analyses were conducted using an explanatory sequential design with focus group discussions (FGDs) to intensively explain the results of the quantitative analyses [25]. In particular, the study focused on FGDs with dental students to elucidate the underlying reasons for the obvious differences in the results obtained by participants studying dentistry and students from other disciplines. The FGDs were conducted at the stated university 5 to 8 months after each IPE workshop attended by the student participants. A total of three FGDs were conducted until no new themes emerged from the discussions (i.e., until saturation was achieved). Purposive sampling was employed for the FGDs, and 11 dental students who appeared to a dental teacher (KN) to be particularly capable of expressing their opinions on the subjects in focus were approached via email and/or face-to-face. Of these students, nine agreed to participate in the study. Snowball sampling was also utilized through these initial participants, and an additional eight students were thus engaged. Ultimately, a total of 17 dental students participated in the FGDs, with each group comprising five to seven students.

The FGDs were conducted using interview guides prepared in advance on the basis of results obtained from quantitative analyses. The dental students were asked their opinions on specific questions during the FGDs. Why were the pre-test scores of the dental students lower than those of the medical and nursing students? Why did the post-workshop scores obtained by dental students not match the tallies attained by the medical or nursing students? How could the IPE workshops be conducted to further enhance the scores of dental students? A medical teacher (NN) conducted one FGD, and another medical instructor (MN) conducted the other FGDs so that it would be easier for the participating dental students to speak candidly about varied aspects of dentistry and dental education. Each FGD lasted approximately 40 minutes, and all three sessions were audio-recorded and subsequently transcribed verbatim.

Deductive coding was performed; to this end, a list of predetermined codes was derived from the key factors identified through the preceding quantitative analyses [25, 26]. The codes were independently applied to the transcripts by two medical teachers (MN, KY) rather than dental educators to enable the observation of concepts that could otherwise be deemed information that was taken for granted as implicitly shared understanding. Such details could be overlooked between students and teachers of dentistry as common knowledge that was understood without saying. Any disagreements between the two coders were resolved through a discussion with another medical teacher (NN). The application of codes introduced during the coding process that differed from the predetermined codes and were deemed useful in explaining the findings of the quantitative analyses was also allowed [26]. The coding was conducted using Word 2016 (Microsoft, Redmond, Washington, USA) to highlight phrases in the transcripts and organize the codebook. Member checking was executed by sending the interpretations of the coded transcribed text by the researchers to ten student participants of the FGDs who had agreed to further contact for the study. The feedback of these ten students was sought to confirm whether the interpretations effected by the researchers reflected the perceptions and ideas of the FGD participant groups. Consequently, the initial interpretation was partially modified to fully resonate with the perceptions and ideas of the participants. In addition, two dental teachers (KN, JT) reviewed the final understanding and deemed it appropriate.

## Results

Table 1 exhibits the characteristics of the study participants. Of the 378 students engaged in this study, 92 (24.3%) were dental students, 190 (50.3%) were medical students, and 96 (25.4%) were nursing students. To clarify the sex distribution, 54.3% of the dental students and 69.5% of the medical students were male, while 97.9% of the nursing students were female.

**Table 1:** Characteristics of study participants.

	Dental students (n = 92)	Medical students (n = 190)	Nursing students (n = 96)
Variable	n (%) or Mean (SD)		
Gender			
Male	50 (54.3)	132 (69.5)	2 (2.1)
Female	42 (45.7)	58 (30.5)	94 (97.9)
Age (years)	24.6 (1.8)	24.2 (2.1)	21.7 (1.4)

Abbreviations: SD = standard deviation.

### Quantitative analyses

Table 2 displays by discipline the total RIPLS scores attained by the participants in the pre- and post- tests. The total RIPLS scores increased significantly for all three disciplines after the IPE workshops: the mean difference was 6.60 (95% CI 4.95 to 8.25; *p* < 0.001) for dental students, 5.06 (95% CI 4.01 to 6.12; *p* < 0.001) for medical students, and 5.19 (95% CI 4.03 to 6.34; *p* < 0.001) for nursing students. No significant differences were discovered in the increases in the total RIPLS tallies after workshops between dental and medical students and between dental and nursing students.

**Table 2:** Pre-test and post-test total RIPLS scores by discipline.

	Dental students	Medical students	Nursing students
Total RIPLS scores	Mean (SE)		
Pre-test	77.0 (0.89)	81.3 (0.67)	82.2 (0.69)
Post-test	83.6 (0.92)	86.4 (0.56)	87.4 (0.61)

Abbreviations: RIPLS = readiness for interprofessional learning scale; SE = standard error.

**Fig 1:**
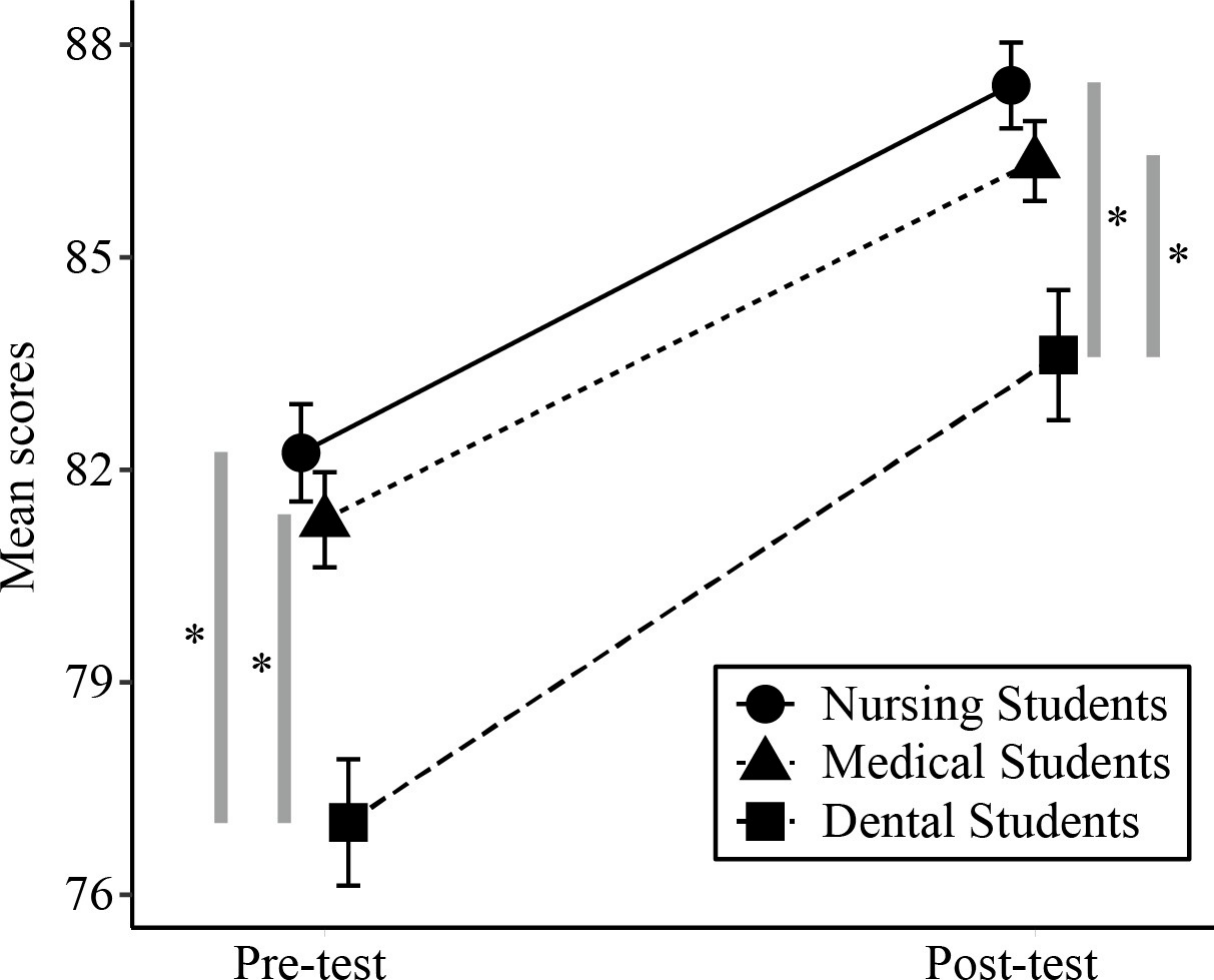
Pre-test and post-test total RIPLS scores. Abbreviations: RIPLS = readiness for interprofessional learning scale. Error bars indicate standard error. Asterisks denote significant differences in the total RIPLS scores between two groups at the time of each survey (*p* < 0.05, Welch’s t-test).

Table 2 and Fig 1 evince that total RIPLS score of the dental students was significantly lower on the pre-test than the marks obtained by medical students (mean difference = 4.27; 95% CI 2.07 to 6.47; *p* < 0.001) and by nursing students (mean difference = 5.22; 95% CI 3.00 to 7.44; *p* < 0.001). Similarly, total RIPLS score of the dental students was significantly lower on the post-test than the tallies attained by the medical students (mean difference = 2.74; 95% CI 0.61 to 4.87; *p* = 0.012) and by the nursing students (mean difference = 3.81; 95% CI 1.63 to 5.98; *p* < 0.001).

### Qualitative analyses

The FGDs yielded three principal themes that could comprehensively explain the results of the quantitative analyses: 1) dental students rarely felt the need for interprofessional collaborations before the IPE workshop, 2) dentists often worked on their own without the need for interprofessional collaborations, and 3) dental students believed their contribution to the IPE workshop was insufficient.

#### 1) Dental students rarely felt the need for interprofessional collaborations before the IPE workshop

The quantitative analyses disclosed that the pre-test scores of the participating dental students were lower than those achieved by the medical and nursing students; thus, the dentistry participants were asked to opine in the FGDs about the reasons for this outcome. They said that before the IPE workshop, there were few occasions when they felt the need for interprofessional collaborations. The patients in the simulated scenarios presented during the workshops suffered systemic problems in addition to their oral ailments, and according to the participating students, such patients were rare in the clinical practice of dentistry to their knowledge. In addition, the dental students stated that they had seldom seen an instance of collaborative treatment or care by multiple healthcare professionals.

One participant (Student 1) asserted, “During the students’ clinical practice, we met very few patients who had systemic problems like those in simulated scenarios.” Another participant of the FGDs (Student 2) said, “During the students’ clinical practice, there were few cases in which we needed to collaborate with healthcare professionals other than dentists.” A third dental student (Student 3) claimed, “I have never seen an actual case in which medical doctors, nurses, and dentists collaborate. I doubt if interprofessional collaboration found in simulated scenarios is carried out in practice… for a single patient.”

#### 2) Dentists often work on their own without the need for interprofessional collaborations

To explain the reasons for the lower pre-test scores attained by dental students, the FGD participants also said that in many of their activities, dentists tended to work on their own without the need for interprofessional collaborations. They estimated that such work-related features pertaining to dentists could have influenced the motivations of dental students. A dental student (Student 4) stated:

Dentists are in charge of almost everything in patient care… Medical students are taught that doctors from various specialties should work together to treat a patient—just as an ophthalmologist finds a goiter and refers the patient to a thyroid specialist. However, we [dental students] are taught that dentists should not only treat cavities on their own but also construct artificial dentures and that those who cannot do so are useless.

#### 3) Dental students believed their contribution to the IPE workshop was insufficient

The results of the quantitative analyses evidenced that the post-workshop scores of dental students did not match the tallies of the medical or nursing students. Thus, the dental students were asked to estimate the reason for this deficiency during the FGDs. In this context, the participants stated that they felt insufficient in terms of their contributions to the workshop activities, especially group discussions. A dental student (Student 5) asserted, “During the workshop, mainly, medical and nursing students contributed [in making decisions about the treatment and care of the patient in the scenario].” Another dental student (Student 3) claimed, “There was little opportunity to express my opinions from a dental perspective… the only opinions I expressed were about oral care…”

When asked to discuss how the IPE workshops should be conducted to enhance the RIPLS scores of dental students, the participants stated that the scenario should not be altered to increase the sense of dental students regarding their contribution because it already encompassed enough material regarding dental problems. One dental student (Student 6) opined, “If the scenario is altered such that there is a higher involvement of dentistry, students from other disciplines would feel like we did [a sense of under-contribution].” Another participant (Student 7) noted, “The scenario itself was not bad.”

One student suggested that the dental students’ sense of contribution could be enhanced if more time and opportunities were accorded to dental students to express their opinions from a discipline-specific perspective because the patients in the given scenarios suffered from substantial dental problems. A dental student (Student 3) explained, “The patients in the scenarios were difficult cases… from a dental perspective… We could have contributed more if we had been assigned the task of presenting a detailed dental treatment plan.”

## Discussion

This study found that the total RIPLS scores of dental, medical, and nursing students increased significantly after attending the IPE workshops. Thus, the IPE workshops applied a positive effect to some extent on students of all three disciplines. However, the dental students scored significantly lower than the students of the other two disciplines in both the pre- and post- workshop RIPLS. The qualitative study revealed the three facets perceived by dental students as rationales for the lower scores.

A prior study conducted an IPE program for dental, medical, and nursing students and reported that the total RIPLS scores of dental and medical students did not increase significantly after the IPE program, but the marks attained by nursing students increased significantly [18]. Haber et al. evaluated the impact of IPE programs by adopting the Interprofessional Collaborative Competency Attainment Scale (ICCAS), another validated scale used to measure interprofessional care competencies [27, 28]. Their study reported significant increases in the mean scores of dental, medical, and nursing students, respectively, after attending IPE programs [27]. The RIPLS scores of dental, medical, and nursing students increased significantly after the IPE workshops in the present study. This amelioration could be attributed to the simulated clinical scenarios prepared to fully utilize the expertise of students of all three disciplines.

The dental students scored significantly lower than the medical and nursing students in the totals achieved in both the pre- and post- workshop RIPLS administrations. Haber et al. reported that the post-test mean ICCAS scores [28] of medical students were significantly lower than the marks attained by dental and nursing students [27]. Haber et al. attributed the lower scores of the medical students to the fact that the main component of the practical study of the participating medical students, who were enrolled in their second year of medical school, was physical examination (i.e., the content of their practice was not related to interprofessional collaboration). These students had not yet begun their clinical clerkship, whereas the participating dental and nursing students were in their final year of study and had already experienced clinical practice [27]. The dental, medical, and nursing students engaged in the present study had already experienced clinical practice; however, the post-test total RIPLS score of the dental students was found to be significantly lower than the marks attained by the medical and nursing students.

The qualitative part of the current study revealed that the dental students felt that even though they had begun experiencing clinical practice as students, they were accorded scant opportunities to realize the need for interprofessional collaborations. The revised version of Biggs’ 3P model suggests that presage factors (the contexts in which instruction occurs and the characteristics of teachers and learners) influence the effectiveness of IPE [8, 16]. Hence, the lesser previously attained awareness of the importance of IPE in dental students vis-à-vis the medical students participating in the present study could explain the differences in outcomes between the previous and current investigations. Congruent with this argument, Hamil asserted that dental students tend to have insufficient exposure to multidisciplinary healthcare [14]. It is, therefore, desirable that dental students are accorded increased opportunities of exposure to interprofessional collaborations by healthcare professionals in their clinical practice or in other settings.

In addition, the results of the qualitative analysis performed for this study reveal that dental students believe that dentists often work on their own without the need for interprofessional collaborations. This outcome is aligned with the implication previously outlined by Hamil that dental students may perceive the environment of dentistry as predominantly a solitary practice [14]. Hamil also asserted that dental educators should help their students understand the critical functions performed by interprofessional collaborations [14]. Thus, educators must proactively teach dental students the importance of interprofessional collaborations even as they inculcate in them the capability of acting on their own in many aspects of their practice.

The insufficient sense of contribution to the workshop was identified by the participating dental students as another theme emerging from the qualitative aspect of this study. They did not believe that the simulated scenarios should be modified to increase their sense of contribution to the workshop. Similarly, a prior study implied that dental-focused topics did not necessarily increase the positive effects of IPE on dental students [29]. Alternatively, the dental students who participated in the present study recommended an increase in the time and opportunities accorded to them to express their opinions from a discipline-specific perspective. The revised Biggs’ 3P model for IPE states that facilitation style is an essential “process factor” [8, 16]. Therefore, these findings suggest that the modification of the workshop facilitation style to offer more time and occasion for students to express discipline-specific opinions would improve the readiness of participants for interprofessional collaboration.

Several limitations of this study must be acknowledged. First, some prior reports have argued that RIPLS has problems in relation to validity [30, 31]. However, although internal consistencies in the subscales were unstable, the internal consistency in the total scores appeared to be satisfactory in most of the translated versions, including the Japanese version, in previous studies [24, 30]. Therefore, we analyzed only the total scores. Second, the social desirability bias may have affected the student responses because the data obtained from the administered RIPLS were self-reported and were collected with the names of the respondents. Third, the findings of this study may not be transferable to other environments because it was conducted at one university. A joint research project with other universities is warranted to confirm the findings of this study. Fourth, this study did not incorporate a long-term follow-up methodology to assess chronological changes in the perceptions and attitudes of participating students. Fifth, this investigation is unable to elucidate the impact of IPE on behavioral changes in workplaces or to enumerate its benefits to patients because it attended to the perceptions and attitudes of students vis-à-vis interprofessional learning as measured through the RIPLS [8]. Sixth, the FGDs were conducted after an interval of five to eight months. However, in general, the students of each discipline strove hard to pass the national qualifying examination during the period. Therefore, we assumed that the students could provide deeper insights about IPE after brushing up on their expertise during the period. Seventh, as the participants in the FGDs were selected, the results of the qualitative analysis might not necessarily represent the opinions of the entire group of dental students. However, we conducted the FGDs with students who we thought would be particularly able to express their opinions on the focus group topics to obtain “rich” data by generating detailed narratives and interactive group content [32]. Furthermore, the FGDs were conducted until saturation was reached, and member checking was conducted to augment the credibility of the results. Finally, this study did not conduct FGDs with medical or nursing students, as the aim of the qualitative analyses was to elucidate reasons for the lower scores attained by dental students in the RIPLS, which is a self-assessment instrument.

The results of this study suggest three possible approaches toward the enhancement of the preparedness of students for interprofessional collaborations: 1) increase their opportunities to attain exposure to interprofessional collaboration in varied settings, 2) proactively teach students the importance of interprofessional collaborations even in environments prone to solitary practices, and 3) provide more time and occasions during the facilitation of IPE workshops so students can express discipline-specific opinions. These methods will aid the development of evidence-based IPE programs and will create environments that enable students from all three disciplines to achieve sufficient levels of interprofessional collaboration. Further research initiatives are required to evaluate whether such evidence-based IPE programs and environments would actually achieve the stated purpose.

## Conclusions

This study found interdisciplinary differences in the preparedness of dental, medical, and nursing students for interprofessional learning. It also elucidated possible factors and reasons for the discovered disparities: dental educational settings offer students insufficient opportunities of exposure to interprofessional collaborations by healthcare professionals, the environments of dentists appear to be solitary with regard to many activities, and dental students felt they did not contribute sufficiently to the activities conducted during the IPE workshops. However, these divergences may be resolved by placing a higher value on interprofessional collaborations in educational environments, enhancing the awareness of educators, and ameliorating the facilitation style of the IPE workshops to allow students to fully utilize their discipline-specific expertise. It is hoped that IPE programs and environments developed on the basis of such experiential evidence would allow students from all disciplines to achieve sufficient levels of interprofessional collaboration and thus ensure the optimal care of prospective patients.
